# Vascular Calcification in Chronic Kidney Disease and Hemodialysis: Pathophysiological Mechanisms and Emerging Biomarkers

**DOI:** 10.3390/medicina61122169

**Published:** 2025-12-05

**Authors:** Marcel Palamar, Iulia Dana Grosu Radulescu, Maria Daniela Tanasescu, Alexandru Sircuta, Flaviu Bob

**Affiliations:** 1 Deva Emergency County Hospital, 330004 Deva, Romania; marcel.palamar@umft.ro; 2 Centre for Molecular Research in Nephrology and Vascular Disease, Faculty of Medicine, “Victor Babeș” University of Medicine and Pharmacy, 300041 Timisoara, Romania; alexandru.sircuta@umft.ro (A.S.); bob.flaviu@umft.ro (F.B.); 3 Department of Internal Medicine II—Nephrology University Clinic, “Victor Babeș” University of Medicine and Pharmacy, 300041 Timisoara, Romania; 4 County Emergency Hospital, 300723 Timisoara, Romania; 5 Nephrology Department, Bucharest Emergency University Hospital, 050098 Bucharest, Romania

**Keywords:** chronic kidney disease, hemodialysis, vascular calcification, mineral bone disorder, matrix Gla protein, osteocalcin, parathyroid hormone, vitamin K deficiency

## Abstract

*Background and Objectives*: Vascular calcification (VC) is a major contributor to cardiovascular morbidity and mortality in patients with chronic kidney disease (CKD), particularly those on hemodialysis. Once considered a passive process, VC is now recognized as an active, cell-mediated pathology influenced by mineral dysregulation, chronic inflammation, and oxidative stress. This review aims to synthesize current evidence on the underlying mechanisms of VC in CKD and hemodialysis, with particular focus on emerging biomarkers and therapeutic implications. *Materials and Methods*: A structured narrative review was conducted by searching PubMed, Web of Science, ScienceDirect, and Google Scholar. The final search was completed on 29 August 2025. A total of 1326 articles were initially retrieved, of which 65 met the inclusion criteria and were analyzed. Studies addressing VC mechanisms, the bone–vascular axis, mineral metabolism, vitamin K–dependent proteins, and biomarkers such as matrix Gla protein (MGP), osteocalcin (OC), and intact parathyroid hormone (iPTH) were included. *Results*: VC in CKD arises from phenotypic transformation of vascular smooth muscle cells, vesicle-mediated calcification, oxidative stress, and impaired activity of endogenous calcification inhibitors. Disruption of the fibroblast growth factor 23 (FGF23)–Klotho axis and secondary hyperparathyroidism further exacerbate vascular pathology. Among emerging biomarkers, dp-ucMGP reflects vitamin K deficiency and correlates with calcification burden, while OC and iPTH provide insight into bone–vascular crosstalk and mineral turnover. However, biomarker interpretation is limited by assay variability, renal clearance, and clinical heterogeneity. *Conclusions*: VC in CKD represents a complex process driven by systemic and cellular dysregulation. While biomarkers such as dp-ucMGP, OC, and iPTH offer mechanistic insights and prognostic potential, further validation is required for clinical application. A multimarker approach, combined with individualized management of mineral metabolism, may improve risk stratification and therapeutic targeting in this high-risk population.

## 1. Introduction

As CKD progresses, its cardiovascular implications become increasingly evident, with risk accelerating in parallel to declining renal function. In clinical cohorts, reductions in eGFR and rises in albuminuria consistently stratify patients toward worse outcomes, including higher rates of hospitalization, cardiovascular events, and progression to kidney failure [1,2,3]. This gradient of risk is particularly pronounced in advanced categories such as G4A3, where the burden of cardiovascular morbidity substantially exceeds that observed in milder stages. Patients undergoing hemodialysis (HD) remain the most vulnerable subgroup, as HD-related hemodynamic stress, inflammation, and uremic toxin exposure further amplify cardiovascular risk [1,4]. In particular, early-stage CKD may remain clinically silent, especially in older adults, yet still confers a disproportionately elevated cardiovascular risk profile, stressing the need for early recognition and aggressive risk factor management.

Among the multiple complications of CKD, cardiovascular disease remains the leading cause of death, particularly in patients undergoing hemodialysis. While traditional risk factors such as hypertension, diabetes, and dyslipidemia are highly prevalent in this population, they do not fully explain the strikingly elevated cardiovascular morbidity and mortality observed [5,6]. CKD-specific mechanisms, including chronic inflammation, oxidative stress, uremic toxin accumulation, and disturbances in mineral metabolism, contribute synergistically to vascular damage.

Vascular calcification (VC), a hallmark of cardiovascular pathology in CKD, has emerged as a highly prevalent, actively regulated, and clinically significant process. Far from being a passive phenomenon, VC involves the osteogenic transformation of vascular smooth muscle cells, vesicle-mediated mineral deposition, and suppression of calcification inhibitors such as matrix Gla protein (MGP) and fetuin-A [7,8,9]. This process is further influenced by the CKD–mineral and bone disorder (CKD–MBD) axis, where imbalances in calcium, phosphate, parathyroid hormone (PTH), and fibroblast growth factor 23 (FGF23) drive both skeletal and vascular pathology [10,11,12].

In patients on dialysis, the burden of vascular and valvular calcification is exacerbated by hemodynamic stress, fluctuating mineral levels, and systemic inflammation. The consequences include arterial stiffness, increased pulse wave velocity, left ventricular hypertrophy, heart failure, and higher rates of myocardial infarction and stroke [8,13,14]. Despite the clinical importance of VC in CKD, its pathophysiology remains incompletely understood, and effective therapeutic interventions remain limited.

Recent research has focused on identifying biomarkers that can reflect the activity of vascular calcification, stratify risk, and guide therapeutic decisions. These include vitamin K–dependent proteins such as MGP and osteocalcin (OC), the inactive form dp-ucMGP, iPTH, and indicators of bone turnover. However, their clinical utility is still evolving, and controversies persist regarding their predictive accuracy, assay standardization, and response to interventions such as vitamin K supplementation or calcimimetics [15,16].

This review aims to provide a comprehensive synthesis of the molecular mechanisms, clinical implications, and emerging biomarkers associated with vascular calcification in CKD and hemodialysis populations. Beyond summarizing pathophysiological pathways, we emphasize the translational relevance of novel biomarkers, particularly vitamin K–dependent proteins, osteocalcin, and iPTH, and evaluate their potential roles in risk stratification and therapeutic guidance.

## 2. Materials and Methods

This review was conducted following the SANRA (Scale for the Assessment of Narrative Review Articles) guidelines to ensure transparency, coherence, and methodological rigor in line with best practices for narrative reviews.

### 2.1. Literature Search Strategy

A structured literature search was performed across four electronic databases: PubMed, Web of Science, ScienceDirect, and Google Scholar. The final search was completed on 29 August 2025.

We used a combination of MeSH and free-text terms. A sample search string was: (“vascular calcification” OR “arterial calcification”) AND (“chronic kidney disease” OR CKD) AND (“mineral bone disorder” OR MBD) AND (“matrix Gla protein” OR MGP OR osteocalcin OR “parathyroid hormone” OR PTH OR FGF23)

Boolean operators (AND, OR) were used to refine search sensitivity and specificity. No restrictions were applied to publication year or geographic location. However, only articles published in English in peer-reviewed journals were included.

### 2.2. Inclusion and Exclusion Criteria

We included studies that met the following criteria:

- Addressed vascular or valvular calcification in the context of chronic kidney disease
- Explored relevant biomarkers, pathophysiological mechanisms, or therapeutic strategies
- Employed human or animal models, clinical or experimental designs, or provided systematic evidence reviews

We excluded:

- Conference abstracts, letters, editorials, and commentaries without primary data
- Articles not directly focused on CKD-related calcification
- Non-English publications and grey literature

### 2.3. Study Selection Process

A total of 1326 records were initially retrieved. After title and abstract screening, 142 full-text articles were assessed for eligibility. Following application of inclusion/exclusion criteria, 65 studies were included in the final synthesis. We also performed manual reference mining of key articles to identify any additional eligible studies. Where screening decisions differed between co-authors, disagreements were resolved by discussion and consensus.

**Figure 1:**
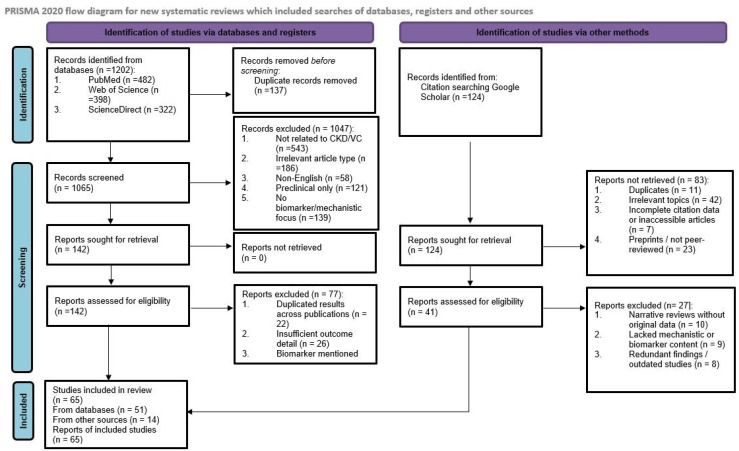
PRISMA 2020 flow diagram summarizing the study selection process.

To enhance transparency, a PRISMA-style flow diagram summarizing the selection process is included (Figure 1).

### 2.4. Assessment of Study Quality and Relevance

Due to the narrative design, no formal risk-of-bias assessment tool was applied. However, preference was given to studies that met the following informal quality benchmarks:

- Recent publications (within the last 10 years)
- Published in high-impact peer-reviewed journals
- Clearly described methodology and clinically relevant conclusions
- We prioritized inclusion of systematic reviews, randomized controlled trials, and well-designed observational studies, supplemented by mechanistic research from experimental models when appropriate.

### 2.5. Ethics

This work involved no new data collection, human participants, or clinical interventions, and therefore did not require ethical approval.

## 3. Results

### 3.1. CKD Progression and Hemodialysis-Related Cardiovascular Risk

CKD is a major and growing global health concern, affecting more than 800 million individuals and ranking among the top ten causes of death worldwide [17,18]. As renal function declines, the risk of adverse cardiovascular outcomes rises significantly, positioning CVD as the leading cause of mortality in CKD patients, particularly those undergoing HD [1,17]. The 2024 KDIGO guidelines reaffirm a comprehensive risk stratification model based on the CGA classification system—incorporating Cause, GFR category, and Albuminuria category—enabling more accurate staging and prognostication [17]. Both a reduced eGFR and elevated albuminuria are independently and synergistically associated with increased risks of hospitalization, cardiovascular events, progression to ESRD, and all-cause mortality—even during early CKD stages [1,2,3]. For example, individuals in the G4A3 category face substantially higher risks compared to those in G1A1 or G2A1, stressing the additive prognostic value of the CGA framework [1]. Nonetheless, early-stage CKD often remains asymptomatic and underdiagnosed, particularly among elderly individuals who may exhibit slower disease progression but possess an inherently higher cardiovascular risk profile [1,17].

Progression from CKD to ESRD introduces additional cardiovascular complications. While HD remains essential for solute and fluid removal in ESRD, it imposes unique physiological stress. HD patients experience disproportionately high rates of cardiovascular morbidity and mortality, driven by a combination of traditional risk factors such as hypertension, diabetes mellitus, and dyslipidemia, and CKD-specific mechanisms. Uremic toxins such as indoxyl sulfate and trimethylamine-N-oxide (TMAO), which are poorly cleared even with dialysis, exert pro-inflammatory and pro-atherogenic effects. These compounds impair endothelial nitric oxide production, compromise vascular function, and promote vascular calcification and atherogenesis [19,20].

Hemodialysis also exacerbates cardiovascular risk through bioincompatibility between blood and dialysis membranes. Contact with synthetic surfaces activates both the complement system, particularly via the alternative and lectin pathways, and the coagulation cascade through factor XII (FXII), leading to generation of pro-inflammatory mediators such as C5a and bradykinin. These mediators promote immune cell recruitment, cytokine release, and endothelial damage, ultimately contributing to chronic vascular inflammation and thrombosis [21].

Complications inherent to CKD such as anemia, mineral and bone disorders (CKD–MBD), and dyslipidemia, further aggravate cardiovascular risk. Anemia, largely caused by insufficient erythropoietin production and iron imbalance, increases cardiac workload and fosters left ventricular hypertrophy. CKD–MBD, involving disturbances in calcium, phosphate, parathyroid hormone (PTH), and fibroblast growth factor 23 (FGF23), facilitates arterial calcification and vascular stiffness, both of which correlate with increased cardiovascular mortality [18]. In addition, dyslipidemia in CKD often features dysfunctional high-density lipoprotein (HDL) and a pro-inflammatory lipid profile.

The hemodynamic instability associated with HD—characterized by rapid fluid shifts and blood pressure fluctuations—further contributes to myocardial damage over time, leading to structural alterations such as myocardial fibrosis and diastolic dysfunction. Emerging data suggest that frailty and abnormal body composition, including sarcopenic obesity, may also reduce cardiovascular resilience in HD patients [22].

These interrelated pathophysiological processes culminate in markedly elevated mortality rates in clinical settings. Observational cohort studies report five-year mortality exceeding 50% among HD patients with preexisting CVD, compared to approximately 24% in those without CVD [23]. A prior history of stroke is identified as the strongest independent predictor of mortality. Furthermore, patients treated with HD exhibit worse survival compared to those receiving peritoneal dialysis (hazard ratio 1.57) [23].

In summary, the existing literature highpoints the critical importance of early identification and management of cardiovascular risk in CKD and ESRD populations. Incorporating GFR and albuminuria into baseline assessments, alongside aggressive control of traditional and CKD-specific risk factors, is vital for mitigating cardiovascular complications. Personalized treatment approaches, appropriate dialysis modality selection, and multidisciplinary care models are also indispensable for optimizing outcomes in this high-risk group.

### 3.2. Pathogenic Pathways Linking Mineral Dysregulation to Vascular and Valvular Calcification in CKD

Vascular and valvular calcification in CKD is an active, tightly regulated process influenced by vascular smooth muscle cell (VSMC) reprogramming, oxidative stress, inflammation, and mineral imbalance. These pathological features are interrelated and provide a mechanistic foundation for cardiovascular risk in patients with CKD and ESRD.

#### 3.2.1. VSMC Phenotypic Transition and Matrix Mineralization

In response to hyperphosphatemia, inflammation, and oxidative stress, VSMCs lose their contractile phenotype and adopt osteoblast-like features, driven by transcription factors such as Runx2, BMP2, and Msx2 [24]. Matrix vesicles (MVs) and apoptotic bodies act as nucleation sites for hydroxyapatite deposition, particularly in the arterial media [25,26]. Chronic stress also dysregulates protective autophagy [27], and activates Wnt/β-catenin, Notch, and NF-κB pathways [28]. These molecular changes promote calcific transformation and extracellular mineralization.

Phosphate entry via PiT-1 and PiT-2 further exacerbates calcification, especially when endogenous inhibitors like MGP and fetuin-A are reduced, as often observed in vitamin K deficiency or phosphate overload [29]. Epigenetic changes, including altered microRNA expression and histone modification, reinforce this pro-calcific state [30], while matrix degradation by MMPs and alkaline phosphatase facilitates hydroxyapatite deposition [31,32].

The key molecular and cellular mechanisms driving vascular calcification are summarized in Table 1.

**Figure 2:**
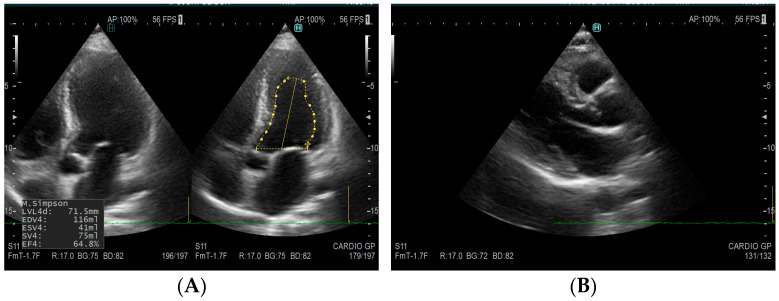
Transthoracic echocardiographic images of normal valves. (**A**) Apical five-chamber echocardiographic view demonstrating normal mitral and aortic valve anatomy. (**B**) Parasternal long-axis echocardiographic view showing normal mitral and aortic valves. Source: Images obtained at Spitalul de Urgență Deva, Romania. Used with institutional permission.

**Figure 3:**
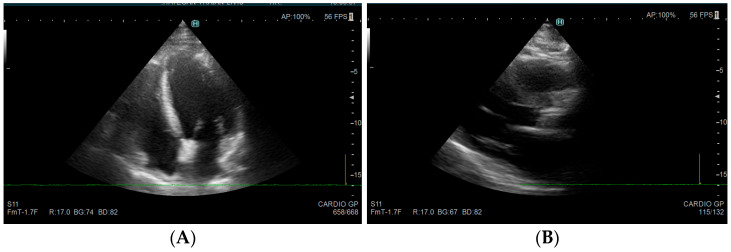
Transthoracic echocardiographic appearance of advanced mitral and aortic valve calcification in chronic kidney disease. (**A**) Parasternal long-axis view showing marked echogenic thickening of the mitral valve leaflets, consistent with severe calcific degeneration. The mobility of the valve is reduced, and acoustic shadowing is present. (**B**) Apical view revealing calcified aortic cusps with restricted opening during systole, indicative of hemodynamically significant aortic stenosis. Source: Images obtained at Spitalul de Urgență Deva, Romania. Used with institutional permission.

Representative echocardiographic findings are presented in Figure 2 and Figure 3.

Figure 2 illustrates representative echocardiographic views of normal mitral and aortic valves. 

Representative echocardiographic images demonstrating severe aortic valve calcification are shown in Figure 3.

#### 3.2.2. Inflammation, Oxidative Stress, and Antioxidant Deficiency

CKD is characterized by systemic inflammation and increased oxidative stress, which together accelerate vascular injury. Reactive oxygen species (ROS), generated by NADPH oxidases and dysfunctional mitochondria, activate NF-κB, leading to cytokine release (e.g., IL-6) and enhanced VSMC osteogenic activity [33,34,35]. Simultaneously, natural antioxidant defenses such as SOD, GPx3, and catalase are often impaired [6,7,8,9,10,11,12,13,14,15,16,17,18,19,20,21,22,23,24,25,26,27,28,29,30,31,32,33,34,35,36,37,38], and trace element deficiencies (zinc, selenium) may further worsen redox imbalance [31].

ROS also increase VSMC sensitivity to phosphate, while inflammation boosts PiT transporter expression, creating a vicious cycle of mineral influx and cellular transformation [39].

#### 3.2.3. Bone–Vascular Crosstalk and Endocrine Dysregulation

The bone–vascular axis in CKD describes the paradox of concurrent vascular calcification and bone demineralization. A central driver is dysregulation of the FGF23–Klotho system. Elevated FGF23 and suppressed Klotho levels in CKD lead to phosphate retention and vascular toxicity [40]. FGF23 directly stimulates myocardial hypertrophy via FGFR4 signaling and promotes inflammation [41,42,43], while Klotho deficiency reduces antioxidant and anti-apoptotic activity, amplifying calcification risk.

Uremic toxins like indoxyl sulfate and p-cresyl sulfate also impair MGP and fetuin-A activity and promote pro-calcific pathways via aryl hydrocarbon receptor (AhR) and NF-κB activation [44]. These toxins induce transcription of BMP2 and Msx2, accelerating osteogenic signaling [45].

#### 3.2.4. CKD-MBD and PTH-Mediated Mineral Dysregulation

Mineral and bone disorder (CKD–MBD) contributes to calcification via disturbances in calcium–phosphate homeostasis and parathyroid hormone (PTH) signaling [46,47]. Secondary hyperparathyroidism, driven by phosphate retention and low calcitriol, promotes high-turnover bone disease and mineral efflux, intensifying vascular calcification [48]. It has to be noted that PTH suppression through calcimimetics or parathyroidectomy can mitigate calcification progression [49].

Because intact PTH alone offers limited diagnostic accuracy, bone turnover markers (BTMs) such as bALP, P1NP, and TRAP5b are used to better characterize turnover states. Elevated bALP, in particular, is strongly associated with vascular calcification and fracture risk [50]. KDIGO recommends iPTH and bALP for routine monitoring, though variability in assays and renal clearance issues affect interpretation [51,52,53].

Therapeutic strategies targeting PTH include vitamin D analogs (calcitriol, paricalcitol), extended-release calcifediol (ERC), and calcimimetics (cinacalcet, etelcalcetide). ERC increases 25D while avoiding hypercalcemia or phosphate overload [54,55]. However, large trials have not consistently demonstrated cardiovascular benefit with PTH suppression [46].

Both high-turnover and adynamic bone disease influence vascular calcification. The former increases calcium–phosphate release; the latter reduces skeletal buffering, allowing extraskeletal mineral deposition [47]. Individualized therapy is essential to maintain balance across the bone–vascular axis.

**Figure 4:**
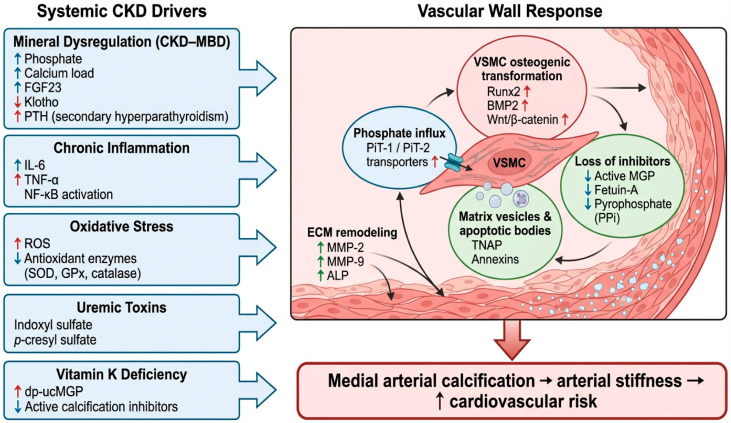
Integrated molecular and cellular mechanisms contributing to vascular calcification in chronic kidney disease. Abbreviations: CKD—Chronic Kidney Disease; VC—Vascular Calcification; VSMC—Vascular Smooth Muscle Cell; FGF23—Fibroblast Growth Factor 23; PTH—Parathyroid Hormone; IL-6—Interleukin-6; TNF-α—Tumor Necrosis Factor-α; ROS—Reactive Oxygen Species; SOD—Superoxide Dismutase; GPx—Glutathione Peroxidase; dp-ucMGP—Dephosphorylated-Uncarboxylated Matrix Gla Protein; BMP2—Bone Morphogenetic Protein 2; PiT-1/2—Phosphate Transporters 1 and 2; TNAP—Tissue Non-Specific Alkaline Phosphatase; MMP-2/9—Matrix Metalloproteinases 2 and 9; PPi—Pyrophosphate; ALP—Alkaline Phosphatase.

To aid visualization of these interacting pathways, Figure 4 summarizes the systemic drivers and vascular wall mechanisms that culminate in medial arterial calcification in CKD.

### 3.3. Biomarkers of Vascular Calcification in CKD

Vascular calcification in CKD represents a progressive process rooted in systemic mineral dysregulation, inflammation, and cellular reprogramming. Biomarkers have emerged as critical tools to understand, monitor, and potentially guide the management of this complication. Among these, vitamin K–dependent proteins (VKDPs), particularly matrix Gla protein (MGP) and osteocalcin (OC), as well as intact parathyroid hormone (iPTH), have shown both pathophysiological and clinical relevance. However, their utility remains nuanced, as each reflects different aspects of the bone–vascular axis and CKD-MBD spectrum.

#### 3.3.1. Vitamin K Deficiency and VKDP Dysregulation in CKD

Vitamin K is an important cofactor for the γ-carboxylation of VKDPs, a modification necessary for their biological activity in coagulation, bone health, and vascular calcification inhibition. Two principal forms exist: vitamin K1 (phylloquinone) and vitamin K2 (menaquinones, especially MK-4 and MK-7). In CKD, functional vitamin K deficiency is highly prevalent, attributed to dietary restrictions, impaired absorption, pharmacologic interference (e.g., sevelamer binding), and increased demand due to vascular injury [15,55].

Subclinical vitamin K deficiency in CKD is reflected by elevated levels of undercarboxylated proteins, particularly dp-ucMGP and ucOC. These forms are inactive and fail to inhibit calcification effectively. Among dialysis patients, the prevalence of functional vitamin K deficiency exceeds 90% when assessed via dp-ucMGP or PIVKA-II (protein induced by vitamin K absence or antagonist II) levels [15].

Despite the biological plausibility, clinical trials evaluating vitamin K supplementation (especially MK-7) in CKD patients have produced limited success. While supplementation consistently reduces dp-ucMGP levels, it has not translated into significant regression of vascular calcification or improved cardiovascular outcomes over short- to mid-term follow-up [56].

#### 3.3.2. Matrix Gla Protein (MGP): A Gatekeeper of Calcification

MGP is a potent endogenous inhibitor of vascular calcification, primarily synthesized by VSMCs and chondrocytes [57]. It antagonizes bone morphogenetic protein-2 and binds calcium salts to prevent hydroxyapatite formation in the vascular wall. For MGP to be fully active, it requires both γ-carboxylation (vitamin K–dependent) and serine phosphorylation. The inactive dp-ucMGP isoform is increasingly recognized as a biomarker of vitamin K deficiency and vascular risk [58].

In CKD and ESRD, circulating dp-ucMGP is frequently elevated due to reduced carboxylation and renal clearance. Clinical studies have demonstrated strong correlations between dp-ucMGP levels, declining renal function, arterial stiffness, and coronary artery calcification (CAC). Moreover, vitamin K supplementation reduces dp-ucMGP levels, confirming its responsiveness to therapy [58,59]. However, its diagnostic precision and prognostic utility in routine nephrology remain under evaluation.

#### 3.3.3. Osteocalcin: Marker of Bone-Vascular Crosstalk

Osteocalcin, another VKDP, is secreted by osteoblasts and exists in both carboxylated (cOC) and undercarboxylated (ucOC) forms, the balance of which is vitamin K–dependent. While traditionally viewed as a marker of bone formation, OC also participates in vascular remodeling, glucose metabolism, and energy regulation [60,61].

In CKD, elevated levels of total OC result from high bone turnover and reduced renal clearance. ucOC has been identified in vascular calcified lesions, co-expressed with osteogenic markers such as Runx2 and bone-specific alkaline phosphatase in transdifferentiated VSMCs [62,63]. This highlights the role of OC not only as a circulating biomarker but also as a local effector of vascular osteogenesis.

Despite the biological relevance, clinical data on OC as a predictive biomarker of vascular calcification are mixed. Some studies associate higher OC levels with increased arterial stiffness, CAC, and cardiovascular mortality, while others attribute these associations to underlying bone turnover rather than direct vascular injury [62,64]. This variability may reflect differences in assay specificity (total vs. cOC vs. ucOC) and heterogeneity in patient populations.

Table 2 outlines the key physiological functions, clinical associations, and diagnostic limitations of osteocalcin in the context of chronic kidney disease.

#### 3.3.4. dp-ucMGP: Surrogate or Specific Marker?

dp-ucMGP has emerged as a promising biomarker linking vitamin K deficiency to VC in CKD. It accumulates in calcifying tissues and correlates with BMP-2 signaling and VC severity [65,66,67,68]. However, its translational value remains limited by key factors. First, its levels are strongly affected by renal clearance, assay type, and comorbid conditions, making cross-study comparisons difficult and interpretation in advanced CKD challenging [68,69,70]. Second, while it is responsive to vitamin K supplementation, its specificity for localized VC—as opposed to systemic vitamin K deficiency—is debated [69,70,71]. Some reports suggest PIVKA-II may outperform dp-ucMGP in certain CKD populations [69].

Despite these issues, dp-ucMGP shows potential as part of multimarker risk models, particularly when combined with other markers such as GDF-15 [72]. Its dynamic modulation by therapy and association with calcification outcomes justify further evaluation, particularly in biomarker-enriched trial designs. Overall, dp-ucMGP is best categorized as a promising but experimental biomarker with limited standalone clinical utility at present.

The following table (Table 3) summarizes the mechanistic features and context-specific clinical challenges of dp-ucMGP in CKD-related vascular calcification, based on key studies.

#### 3.3.5. iPTH: Marker and Modulator

iPTH has a dual role in CKD, as both a mediator of mineral dysregulation and a biomarker of systemic remodeling. Elevated iPTH contributes to high-turnover bone disease, increases circulating calcium-phosphate load, and indirectly promotes vascular calcification [73,74,75]. Conversely, excessive suppression leads to adynamic bone disease, reducing skeletal buffering and enhancing extraskeletal mineral deposition [76].

Clinical studies consistently link extremes of iPTH with poor cardiovascular outcomes and vascular calcification in dialysis patients [77,78,79]. Therapeutic strategies using vitamin D analogues and calcimimetics (e.g., cinacalcet, etelcalcetide) aim to titrate iPTH into an optimal range to mitigate both skeletal and vascular complications [80]. However, heterogeneity in PTH metabolism, assay variability, and lack of consensus on therapeutic targets limit its standalone utility [81].

iPTH is best interpreted in conjunction with other parameters, such as calcium, phosphate, and bone turnover markers, within an integrated CKD-MBD assessment framework.

#### 3.3.6. Biomarkers in Risk Stratification and Prognosis

Beyond pathophysiology, biomarkers like dp-ucMGP, OC, iPTH, and FGF23 are increasingly employed in risk stratification. Multimarker models combining mineral metabolism indicators with cardiac troponins, natriuretic peptides, and imaging findings (e.g., coronary artery calcium score) enhance prognostic accuracy for mortality and cardiovascular events [82,83].

In dialysis populations, such strategies enable identification of high-risk individuals, inform therapy selection, and guide monitoring intensity. However, for routine implementation, assay standardization, outcome validation, and integration into clinical decision pathways remain necessary steps.

Table 4 summarizes the comparative biological relevance, limitations, and clinical readiness of the most frequently studied biomarkers in CKD-associated vascular calcification.

## 4. Discussion

Vascular calcification in CKD represents a convergence point of several systemic and cellular pathologies that challenge conventional paradigms in nephrology, cardiovascular medicine, and endocrinology. Its prominence as a predictor of mortality in CKD, particularly in dialysis patients, has shifted the clinical conversation from traditional cardiovascular risk factors to mechanisms more specific to the uremic milieu, such as mineral bone disorder, inflammation, and endocrine dysregulation [6,8]. The evolving understanding of VC as an actively regulated, osteogenic-like process has reframed it from a passive consequence of aging or atherosclerosis into a dynamic, targetable pathology [7,9,26].

This transformation in conceptual framework mirrors similar shifts seen in other chronic inflammatory conditions, where cellular phenotypes adapt maladaptively to environmental stressors. In VC, the phenotypic switch of vascular smooth muscle cells into osteoblast-like cells is emblematic of this maladaptation, triggered by mineral imbalances, oxidative stress, and uremic toxins [22,24,27,42]. These insights, largely informed by advances in molecular biology and systems medicine, align with a broader trend in chronic disease research that views tissue-specific pathology through the lens of systemic dysfunction.

Emerging biomarkers, such as dp-ucMGP, osteocalcin, and iPTH, offer a glimpse into the complex crosstalk between bone and vasculature [57,58,60,73]. However, they also expose a gap between mechanistic discovery and clinical application. Variability in assay performance, lack of standardized thresholds, and overlapping influences from bone turnover and renal clearance present challenges to integrating these biomarkers into predictive models or treatment algorithms [64,69,70,71].

In this context, the “bone–vascular axis” is not just a metaphor but a physiological reality. As CKD disrupts endocrine regulators like FGF23 and Klotho, the traditional separation between bone metabolism and vascular health dissolves [10,12,40,41]. This convergence creates therapeutic dilemmas: for instance, strategies that reduce PTH may improve bone health but inadvertently impair vascular protection, and vice versa [46,47,51]. Balancing skeletal and cardiovascular outcomes is therefore not only a pharmacologic challenge but a philosophical one, demanding a shift from disease-specific silos to integrative care models.

From a therapeutic standpoint, current strategies remain largely supportive or palliative. Despite clear mechanistic targets like vitamin K pathways, phosphate transporters, or calcification inhibitors, clinical trials have yet to translate these into consistent patient benefit [15,16,56,72]. For example, vitamin K supplementation has failed to demonstrate consistent improvement in clinical VC outcomes, potentially due to short follow-up durations, inadequate dosing, differences in baseline vitamin K status, and lack of patient stratification based on baseline dp-ucMGP levels. To address this, future randomized controlled trials should consider enrichment strategies that incorporate biomarkers like dp-ucMGP to identify subpopulations most likely to benefit. By selecting patients with elevated dp-ucMGP or low vitamin K status, RCTs may increase their likelihood of detecting clinically meaningful effects. Moreover, metalloproteinases, particularly MMP-2 and MMP-9, have emerged as key players in vascular remodeling and calcification in CKD patients. Their activity contributes to extracellular matrix degradation and vascular stiffness, facilitating calcific deposition. Andreucci et al. [83] highlighted the link between MMP dysregulation and aortic aneurysm progression in CKD, suggesting a broader role for MMPs in vascular pathology beyond calcification alone. This adds a layer of complexity to VC pathogenesis and highlights potential new therapeutic targets.

Looking ahead, the integration of VC biomarkers into composite scoring systems, alongside imaging metrics like coronary artery calcium and functional assessments like pulse wave velocity, may offer a path forward [59,65,84]. However, the lack of standardization in assays, particularly for dp-ucMGP, remains a significant barrier. Differences in assay sensitivity, reference ranges, and clinical interpretation hinder cross-study comparisons and regulatory acceptance. International harmonization efforts are needed to standardize biomarker measurement, enable pooled analyses, and guide treatment thresholds.

Furthermore, artificial intelligence (AI) and machine learning (ML) tools hold promise in enhancing VC risk prediction. These technologies can integrate biochemical markers, imaging features, and clinical data to generate individualized risk profiles and uncover hidden patterns not apparent through traditional statistical approaches. AI-driven models could aid clinicians in decision-making by identifying high-risk patients and suggesting targeted interventions, thus facilitating precision nephrology.

In summary, bridging the gap between pathophysiologic insights and clinical translation in VC management requires complex strategies: standardized biomarker platforms, targeted RCT designs using enrichment strategies, incorporation of MMP biology, and novel AI-supported risk models. Such innovations can pave the way for a more personalized, predictive, and preventive approach to vascular calcification in CKD.

## 5. Conclusions

Vascular calcification in chronic kidney disease remains a clinically significant complication that bridges dysregulated mineral metabolism with accelerated cardiovascular risk. While considerable progress has been made in elucidating the molecular mechanisms underlying this process, including the role of vitamin K–dependent proteins such as matrix Gla protein and endocrine markers like iPTH, challenges persist in translating these insights into routine clinical practice. A nuanced understanding of the bone–vascular axis continues to evolve, revealing both the limitations of current surrogate markers and the complexity of their interactions within the uremic milieu. Rather than relying on single biomarkers, future research must refine how we integrate multiple physiological indicators into actionable clinical tools. This includes not only identifying who may benefit from targeted interventions, but also defining when and how such strategies should be implemented for maximal patient impact.

Ultimately, improving outcomes in this high-risk population will depend not just on mechanistic discovery, but on rigorous validation of biomarkers within frameworks that prioritize precision, accessibility, and clinical applicability.

## Figures and Tables

**Table 1:** Molecular and Cellular Drivers of Vascular Calcification.

Mechanism	Key Molecules	Pathway/Effect
Osteogenic reprogramming [24,28]	Runx2, Osterix, BMP2, Msx2	Induces VSMC transition into osteoblast-like phenotype
Epigenetic regulation [30]	miR-125b, miR-30b/c, miR-34a, DNA methylation	Modulate transcriptional control of osteogenic genes
Extracellular vesicles [25]	Annexins, TNAP, Sortilin	Nucleation of hydroxyapatite in MVs and apoptotic bodies
Apoptosis [26]	ROS, mitochondrial dysfunction	Release of apoptotic bodies, trigger calcification sites
Autophagy [27]	mTOR, lysosomal enzymes	Protective at baseline; dysregulation promotes calcification
Redox imbalance [24]	NADPH oxidase, GPx3, SOD, Catalase	Oxidative stress promotes NF-κB and osteogenic signaling
Inflammatory pathways [28]	IL-6, TNF-α, NF-κB	Cytokines enhance VSMC osteogenesis and ECM remodeling
Matrix remodeling [31,32]	MMP-2, MMP-9, Elastase, ALP	Degradation of ECM permits mineral deposition
Signaling pathways [28]	Wnt/β-catenin, Notch, TGF-β/SMAD	Activate calcification genes and matrix deposition
Mineral homeostasis [29]	PiT-1, PiT-2, Pyrophosphate, TNAP	Phosphate influx and PPi degradation accelerate calcification
Bone–vascular axis [29]	FGF23, Klotho, Sclerostin	Endocrine misregulation links bone and vascular calcification
Toxin-activated pathways [29]	Indoxyl sulfate, p-cresyl sulfate, AhR	Promote oxidative, inflammatory, and osteogenic pathways
Inhibitor deficiency [29,31,32]	Fetuin-A, MGP, PPi	Loss of inhibitors removes restraint on mineral growth

**Abbreviations**: AhR—Aryl Hydrocarbon Receptor; ALP—Alkaline Phosphatase; BMP2—Bone Morphogenetic Protein 2; DNA—Deoxyribonucleic Acid; ECM—Extracellular Matrix; FGF23—Fibroblast Growth Factor 23; GPx3—Glutathione Peroxidase 3; IL-6—Interleukin-6; MGP—Matrix Gla Protein; miR—MicroRNA; mTOR—Mammalian Target of Rapamycin; MMP-2/9—Matrix Metalloproteinases 2 and 9; MV—Matrix Vesicle; NADPH—Nicotinamide Adenine Dinucleotide Phosphate (Reduced Form); NF-κB—Nuclear Factor Kappa-Light-Chain-Enhancer of Activated B Cells; Notch—Neurogenic Locus Notch Homolog Pathway; Osterix—SP7 Transcription Factor; PiT-1/2—Phosphate Transporters 1 and 2; PPi—Inorganic Pyrophosphate; ROS—Reactive Oxygen Species; Runx2—Runt-Related Transcription Factor 2; SOD—Superoxide Dismutase; TGF-β—Transforming Growth Factor Beta; TNAP—Tissue Non-Specific Alkaline Phosphatase; TNF-α—Tumor Necrosis Factor-α; VSMC—Vascular Smooth Muscle Cell; Wnt—Wingless/Integrated Signaling Pathway.

**Table 2:** Clinical Roles and Limitations of Osteocalcin in CKD.

Dimension	Description	Implication in CKD
Primary source [60,61]	Produced by osteoblasts; secreted in carboxylated and undercarboxylated forms	Reflects bone formation status and vitamin K availability
Bone matrix role [60]	cOC binds calcium and hydroxyapatite for bone mineralization	Marker of effective skeletal function
Endocrine function [60,64]	ucOC regulates glucose metabolism, insulin sensitivity, and energy homeostasis	May influence vascular health indirectly via metabolic effects
Local vascular expression [63]	Detected in vascular smooth muscle cells undergoing osteogenic transformation	Supports concept of active, cell-driven vascular calcification
Elevated in CKD [62,64]	Total OC levels increased due to high bone turnover and reduced renal clearance	Useful as a marker of CKD–MBD and skeletal pathology
Association with VC [62,63,64]	Higher OC levels correlate with arterial stiffness, CAC, valvular calcification, and CV mortality in dialysis patients	OC may serve as both marker and modulator of vascular pathology
Assay Variability [64]	Total vs. cOC vs. ucOC measured inconsistently across studies	Limits interpretability and cross-study comparison
Confounding factors [64]	OC elevation may reflect bone turnover, not necessarily direct vascular damage	Interpretation must consider CKD–MBD context
Complementary markers [60,63]	MGP, bone-specific ALP, PTH	OC improves risk stratification when integrated in biomarker panels
Clinical utility [64]	Prognostic value debated; remains investigational	Promising, but needs assay standardization and outcome validation

Abbreviations: ALP—Alkaline Phosphatase; CAC—Coronary Artery Calcification; CKD—Chronic Kidney Disease; CKD–MBD—Chronic Kidney Disease–Mineral and Bone Disorder; CV—Cardiovascular; cOC—Carboxylated Osteocalcin;; ESRD—End-Stage Renal Disease; MGP—Matrix Gla Protein; OC—Osteocalcin; PTH—Parathyroid Hormone; ucOC—Undercarboxylated Osteocalcin; VC—Vascular Calcification.

**Table 3:** Mechanistic and Clinical Features of dp-ucMGP in CKD-Associated Vascular Calcification.

Domain	Key Findings	Clinical Implications
Molecular identity [58]	Inactive MGP form lacking γ-carboxylation and phosphorylation	Reflects vitamin K deficiency; lacks calcification-inhibiting activity
Tissue localization [65,66]	Accumulates in vascular lesions and atheroma	Associated with areas of active calcification
Association with VC [65,70]	Correlates with VC severity and CKD progression	Tracks calcification burden in cross-sectional studies
Responsiveness to therapy [67,72]	Decreases with vitamin K supplementation	Useful for monitoring therapeutic effects in interventional trials
Diagnostic specificity [69,71]	Elevated by systemic vitamin K deficiency and renal clearance factors	May not reflect localized vascular pathology alone
Risk model utility [72]	Enhances CAC prediction when used with GDF-15	Valuable as part of multimarker predictive panels
Assay limitations [69]	Non-standardized methods (ELISA vs. CLIA); inter-assay variability	Limits comparability across studies and hinders clinical translation

Abbreviations: dp-ucMGP, dephosphorylated uncarboxylated matrix Gla protein; MGP, matrix Gla protein; BMP-2, bone morphogenetic protein 2; VC, vascular calcification; CKD, chronic kidney disease; CAC, coronary artery calcification; GDF-15, growth differentiation factor-15; ELISA, enzyme-linked immunosorbent assay; CLIA, chemiluminescent immunoassay.

**Table 4:** Comparative Clinical Utility and Readiness of Biomarkers in CKD-Associated Vascular Calcification.

Biomarker	Biological Role	Clinical Associations	Limitations	Strength of Evidence	Readiness Level
dp-ucMGP	Inactive form of MGP; reflects vitamin K deficiency and impaired calcification inhibition [58,65]	Correlates with CAC, arterial stiffness, VC severity in CKD [65,66,67,68]	Affected by renal clearance, assay type, systemic vitamin K status; specificity limited [68,69,70,71]	Modifiable by vitamin K; enhances CAC prediction in models with GDF-15 [72]	Promising, not yet validated for standalone use
Osteocalcin (OC)	Secreted by osteoblasts; ucOC involved in vascular remodeling and metabolic regulation [60,61]	Linked to arterial stiffness, CAC, and CV mortality in some studies [62,63,64]	Assay variability (total, ucOC, cOC); influenced by bone turnover more than VC [64]	Biologically plausible, but prognostic value debated and heterogeneous across cohorts [62,63,64]	Experimental/Promising, requires standardization
iPTH	Regulates calcium-phosphate balance and bone turnover [73,74,75]	Extremes associated with VC, SHPT, CV risk in dialysis [77,78,79]	Not specific to VC; high assay variability; therapeutic targets unclear [81]	Supported by clinical use and observational studies; targeted by calcimimetics and vitamin D analogues [80]	Established, but best used in multimarker context

Abbreviations: dp-ucMGP, dephosphorylated uncarboxylated matrix Gla protein; MGP, matrix Gla protein; OC, osteocalcin; ucOC, undercarboxylated osteocalcin; cOC, carboxylated osteocalcin; iPTH, intact parathyroid hormone; VC, vascular calcification; CKD, chronic kidney disease; CAC, coronary artery calcification; CV, cardiovascular; SHPT, secondary hyperparathyroidism; GDF-15, growth differentiation factor-15.

## Data Availability

No new data were created or analyzed in this study. Data sharing is not applicable.

## Abbreviations

The following abbreviations are used in this manuscript:

BMP	Bone Morphogenetic Protein
CAC	Coronary Artery Calcification
CKD	Chronic Kidney Disease
CKD–MBD	Chronic Kidney Disease–Mineral and Bone Disorder
cOC	Carboxylated Osteocalcin
CVD	Cardiovascular Disease
dp-ucMGP	Dephosphorylated-Uncarboxylated Matrix Gla Protein
ECM	Extracellular Matrix
eGFR	Estimated Glomerular Filtration Rate
ESRD	End-Stage Renal Disease
FG23	Fibroblast Growth Factor 23
FXII	Coagulation Factor XII
GFR	Glomerular Filtration Rate
HD	Hemodialysis
HDL	High-Density Lipoprotein
iPTH	Intact Parathyroid Hormone
MGP	Matrix Gla Protein
MMP	Matrix Metalloproteinase
MVs	Matrix Vesicles
OC	Osteocalcin
PTH	Parathyroid Hormone
ROS	Reactive Oxygen Species
SHPT	Secondary Hyperparathyroidism
TMAO	Trimethylamine-N-oxide
TNF-α	Tumor Necrosis Factor Alpha
ucOC	Undercarboxylated Osteocalcin
VC	Vascular Calcification
VKDPs	Vitamin K–Dependent Proteins
VSMCs	Vascular Smooth Muscle Cells
